# Poster Session II - A247 DIETARY CARRAGEENANS FUEL INFLAMMATION AND IMPAIR EPITHELIAL BARRIER IN SELECT INFLAMMATORY BOWEL DISEASE PATIENTS

**DOI:** 10.1093/jcag/gwaf042.247

**Published:** 2026-02-13

**Authors:** H Gorman, A Voisin, H Olof, O Fedorova, F Moreau, C Bernstein, S R Shaffer, K Chadee, H Armstrong

**Affiliations:** University of Calgary, Calgary, AB, Canada; University of Calgary, Calgary, AB, Canada; University of Manitoba, Winnipeg, MB, Canada; University of Manitoba, Winnipeg, MB, Canada; University of Calgary, Calgary, AB, Canada; University of Manitoba, Winnipeg, MB, Canada; University of Manitoba, Winnipeg, MB, Canada; University of Calgary, Calgary, AB, Canada; University of Alberta, Edmonton, AB, Canada

## Abstract

**Background:**

Dietary carrageenans are thought to contribute to inflammatory bowel disease (IBD), increasing epithelial permeability and promoting inflammation, but studies have demonstrated conflicting evidence to date. Carrageenans are common food additives derived from red seaweed. *In vitro* and animal model evidence suggest that carrageenans may promote inflammatory response, altering the gut microbiome and mucosal thickness, disrupting the gut epithelial barrier, and activating innate immune pathways. Here, we investigated how carrageenan exposure in cell lines, human IBD biopsy-derived organoids, and human tissue explant cultures, affects inflammatory signalling, immune activation, and epithelial tight junction integrity and permeability.

**Aims:**

The specific aims are to examine the impacts of λ, κ, or ι carrageenans on: 1) barrier function, and 2) immune responses.

**Methods:**

T84 and Caco-2 epithelial cell lines and organoids derived from human colonic biopsies from IBD and non-IBD patients were used to interrogate barrier function and wound healing in response to λ, κ, or ι carrageenan (1mg/mL) using transepithelial electrical resistance (TEER) and FITC-dextran permeability assay, western blotting of cell protein lysates, scratch-wound assays, and Hematoxylin and Eosin (H&E) staining of organoid monolayers. THP-1 macrophages and human tissue explants were cultures with carrageenans to evaluate immune response using multi-plex ELISA and immunohistochemistry.

**Results:**

Addition of λ, κ, or ι carrageenan resulted in significantly lower TEER as compared to untreated controls, suggesting reduced barrier integrity. This response was exaggerated in organoids derived from active UC patients. Despite the alteration in TEER, there was no significant difference in tight junction proteins including (e.g., Claudin, Occludin). Wound healing was impaired by λ and κ carrageenan but not ι carrageenan. Organoids showed altered cell morphology and reduced crypt heights. All carrageenans induced pro-inflammatory cytokine secretion and immune activation in select gut biopsy explant cultures, although personalized responses were uncovered.

**Conclusions:**

These findings demonstrate that carrageenans can induce immune activation and inflammation, along with altering epithelial barrier integrity. This highlights the importance of understanding the effects of commonly consumed additives and warrants further investigation into the factors involved in personalized response to carrageenans.

**Funding Agencies:**

CIHRWeston Family Foundation

